# Dissociated coupling between cerebral oxygen metabolism and perfusion in the prefrontal cortex during exercise: a NIRS study

**DOI:** 10.3389/fphys.2023.1165939

**Published:** 2023-07-25

**Authors:** Mikio Hiura, Akio Funaki, Hirohide Shibutani, Katsumi Takahashi, Yoichi Katayama

**Affiliations:** ^1^ Center for Brain and Health Sciences, Aomori University, Aomori, Japan; ^2^ Faculty of Sociology, Aomori University, Aomori, Japan; ^3^ Faculty of Creative Engineering, Kanagawa Institute of Technology, Atsugi, Japan

**Keywords:** cerebral oxygenation, cerebral metabolic rate for oxygen, cerebral blood volume, cerebral blood flow, exercise intensity, postexercise hypotension, mood status

## Abstract

**Purpose:** The present study used near-infrared spectroscopy to investigate the relationships between cerebral oxygen metabolism and perfusion in the prefrontal cortex (PFC) during exercises of different intensities.

**Methods:** A total of 12 recreationally active men (age 24 ± 6 years) were enrolled. They performed 17 min of low-intensity exercise (ExL), followed by 3 min of moderate-intensity exercise (ExM) at constant loads. Exercise intensities for ExL and ExM corresponded to 30% and 45% of the participants’ heart rate reserve, respectively. Cardiovascular and respiratory parameters were measured. We used near-infrared time-resolved spectroscopy (TRS) to measure the cerebral hemoglobin oxygen saturation (ScO_2_) and total hemoglobin concentration ([HbT]), which can indicate the cerebral blood volume (CBV). As the cerebral metabolic rate for oxygen (CMRO_2_) is calculated using cerebral blood flow (CBF) and ScO_2_, we assumed a constant power law relationship between CBF and CBV based on investigations by positron emission tomography (PET). We estimated the relative changes in CMRO_2_ (rCMRO_2_) and CBV (rCBV) from the baseline. During ExL and ExM, the rate of perceived exertion was monitored, and alterations in the subjects’ mood induced by exercise were evaluated using the Profile of Moods Scale-Brief.

**Results:** Three minutes after exercise initiation, ScO_2_ decreased and rCMRO_2_ surpassed rCBV in the left PFC. When ExL changed to ExM, cardiovascular variables and the sense of effort increased concomitantly with an increase in [HbT] but not in ScO_2_, and the relationship between rCMRO_2_ and rCBV was dissociated in both sides of the PFC. Immediately after ExM, [HbT], and ScO_2_ increased, and the disassociation between rCMRO2 and rCBV was prominent in both sides of the PFC. While blood pressure decreased and a negative mood state was less prominent following ExM compared with that at rest, ScO_2_ decreased 15 min after exercise and rCMRO_2_ surpassed rCBV in the left PFC.

**Conclusion:** Dissociated coupling between cerebral oxidative metabolism and perfusion in the PFC was consistent with the effort required for increased exercise intensity and associated with post-exercise hypotension and altered mood status after exercise. Our result demonstrates the first preliminary results dealing with the coupling between cerebral oxidative metabolism and perfusion in the PFC using TRS.

## 1 Introduction

Near-infrared spectroscopy (NIRS) has been used to explore alterations in oxygenation in the prefrontal cortex (PFC) evoked by exercise. Although only a small volume of oxygenation in the PFC is detected by NIRS, its informative index has been assumed to be a surrogate for brain activity during dynamic exercise. Notably, the PFC is involved in the executive control network for autonomic regulation, planning, and action monitoring during exercise, and in the emotional function, including mood status (Davidson, 2003; Heimer and Van Hoesen, 2006). As physiological activity in the brain is accompanied with oxygen consumption for oxidative phosphorylation and glycolysis without oxygen consumption (Raichle and Mintun, 2006), the balance between oxygen delivery and extraction is reflected in the correlation between cerebral blood flow (CBF) and cerebral metabolic rate of oxygen (CMRO_2_). Ever since the relationship between the CBF and CMRO_2_ was first investigated, it was suggested that changes in CBF must involve a tight coupling between cellular energy requirements and the supplies of glucose and oxygen (Roy and Sherrington, 1890). However, neuroimaging studies by positron emission tomography (PET) demonstrated the unexpected finding that the magnitude of increase in CMRO_2_ was much lower than that in CBF during simple somatosensory stimulation (Fox and Raichle, 1986; Fox et al., 1988; Madsen et al., 1999). Subsequent studies postulated regional and activation-dependent differences in the relationship between CBF and CMRO_2_ (Mintun et al., 2002; Vafaee and Gjedde, 2004; Vafaee et al., 2012). To clarify this relationship using a simplified method, CBF and CMRO_2_ have been observed simultaneously. However, obtaining readings of these parameters using only a single PET measurement is impossible. Previous studies quantified CBF and oxygenation in the PFC using a single session of simultaneous measurements of PET and NIRS during hyper- and hypocapnia (Rostrup et al., 2002), acetazolamide administration that induced an increase in hemodynamic aspects (Ohmae et al., 2006) and hemodialysis (Polinder-Bos et al., 2020). Although these studies demonstrated correlations between NIRS variables and CBF or cerebral blood volume (CBV), the relationship between CBF and CMRO_2_ was not described. Furthermore, simultaneous PET and NIRS measurements during exercise have not been reported to date.

During exercise, changes in oxygenation can be measured by NIRS in a restricted volume of the cerebral cortex, but CBF cannot be measured. Accordingly, PET measurements can be used to investigate the relationship between cerebral oxygenation and CBF. However, during exercise, PET measurements are limited as follows: 1) the regional CBF (rCBF) is available as data averaged over 2 min during a period of stability, and 2) the exercise intensity must be low-to-moderate so that head movement does not prevent measurement. Considering these limitations in PET measurement, we demonstrated changes in CBF during and after low-intensity cycling exercise using PET (Hiura et al., 2014; Hiura et al., 2018a). Additionally, we compared changes in oxygenation with absolute values of CBF measured by NIRS and PET, which were measured with a similar exercise protocol but in different groups (Hiura et al., 2018b). Since continuous-wave (CW) NIRS was applied in that study, we could not obtain cerebral hemoglobin oxygen saturation (ScO_2_) and the concentration of total hemoglobin ([HbT]), which indicates CBV, or demonstrate CMRO_2_. In this study, we applied near-infrared time-resolved spectroscopy (TRS) to measure the absolute values of ScO_2_ and [HbT]. CBV can be calculated using [HbT], the molecular weight of hemoglobin, brain tissue density, and blood hemoglobin concentration (HGB) (Ijichi et al., 2005; Roche-Labarbe et al., 2010).

TRS can continuously and simultaneously measure the absolute values of oxyhemoglobin [HbO] and deoxyhemoglobin [HbR] in the cerebral tissue using ScO_2_ as the ratio of [HbO] to [HbT], where [HbT] is the sum of [HbO] and [HbR] (Ohmae et al., 2006; Torricelli et al., 2014; Auger et al., 2016). Based on the Fick principle, CMRO_2_ can be calculated as the product of CBF, HGB, and the oxygen extraction fraction (OEF), which is equal to the arteriovenous oxygen difference, i.e., the difference between SaO_2_ and venous hemoglobin saturation (SvO_2_). As TRS measures ScO_2_ within mixed structures consisting of arteries, capillaries, and vein, the ratio among these components during exercise has not been reported. Accordingly, we assumed that cerebral tissue [Hb] consists of 25% arterial and 75% venous blood [Hb] (Watzman et al., 2000), and SvO_2_ can be calculated using SaO_2_ and ScO_2_. Based on the estimated relationship between CBV and CBF described in previous studies using PET and NIRS (Ito et al., 2001; Brown et al., 2003; Ito et al., 2003; Roche-Labarbe et al., 2010), CMRO_2_ can be estimated using CBV, HGB, and ScO_2_ by TRS. Furthermore, because of the enhanced depth discrimination of absorption changes, TRS provides better attenuation of extra-cerebral effects on measurements than CW NIRS (Ayaz et al., 2022).

The aim of our study was to examine the relationship between cerebral oxygen metabolism and perfusion in the PFC during continuous cycling exercise at low (ExL) and moderate intensities (ExM) at various time points in response to exercise. With this exercise protocol, we intended to induce the augmented sense of effort at ExM and postexercise hypotension (PEH) in which the blood pressure (BP) decreases to a level below the baseline after exercise termination. The cycling exercise was performed in the supine position to compare CBV obtained by TRS with CBF evaluated in the PFC during exercise, with a protocol similar to that in previous studies (Hiura et al., 2018a). The second aim of our study was to clarify the specific role of the PFC in the relationship between cerebral oxygen metabolism and perfusion at various time points in response to exercise. We hypothesized that the relationship between cerebral oxygen metabolism and perfusion in the PFC would change during exercise depending on exercise intensity and the time course. Since the exercise protocol in the present study is associated with an improved mood status (Bartholomew et al., 2005), we also expected that these relationships in the PFC, which involved the executive control network for emotion (Davidson, 2003; Heimer and Van Hoesen, 2006), would change after the exercise termination.

## 2 Materials and methods

### 2.1 Study design and participants

We investigated oxygenation and CBV in the PFC by TRS using the same protocol that was used in the previous PET study, where CBF in the PFC did not significantly change during low-intensity cycling exercise (Hiura et al., 2018a). For characteristics of participants to be consistent with those in the previous study, a total of 12 recreationally active men (mean ± SD age, 26 ± 7 years; body weight, 70.1 ± 7.1 kg; height, 175.1 ± 4.2 cm) participated in the study. None of the participants had a personal history of physical or psychiatric illness or substance abuse, and none were taking any medications. The participants were instructed to live and eat as normally as possible but avoid rigorous exercise, alcohol, and drugs during the 24 h preceding the experimental sessions.

All participants provided written informed consent after a detailed explanation of the study. The study protocol was designed in accordance with the guidelines of the national government and the 2008 revision of the Declaration of Helsinki. The protocol was approved by the ethics committee of the Faculty of Sociology, Aomori University (no. 01-2020).

### 2.2 Experimental protocol

**FIGURE 1:**
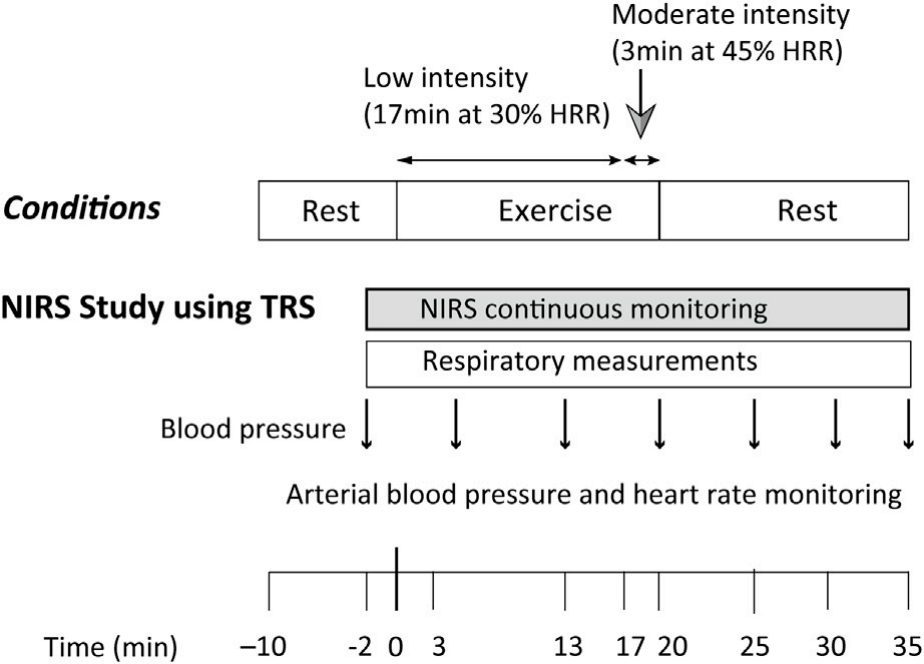
Experimental protocol used for the acquisition of cerebral oxygenation by NIRS during and after a 20-min constant load cycling protocol, comprising 17 min of low-intensity and 3 min of moderate-intensity exercise. The time of exercise initiation was defined as time 0. HRR: heart rate reserve; NIRS: near-infrared spectroscopy; TRS: near-infrared time-resolved spectroscopy.

The protocol was consistent with that of a previous PET study that investigated CBF during ExL, and PEH was evoked after ExM (Hiura et al., 2018a). We followed this protocol so that we could compare CBV measured by TRS during ExL with CBF evaluated in the PET study. A schematic representation of the experimental procedure is shown in Figure 1. The study protocol consisted of a 20-min ergometer cycling protocol under constant load, with 17 min of low-intensity exercise (ExL), followed by 3 min of moderate-intensity exercise (ExM). The 3 min of ExM was used to attain PEH. The participants performed the exercise using a supine ergometer (Angioergo, Lode, Groningen, Netherlands). The work rate (WR) for ExL was set such that HR increased and remained around 30% of a participant’s heart rate reserve (HRR), calculated using the following formula: [(estimated maximum heart rate [HR]−resting HR) × 0.3] + resting HR (Karvonen et al., 1957). The maximum HR (HR_max_) was estimated by subtracting the participant’s age from 220 years. For ExM, the WR was increased by 50% of the ExL level, yielding an increase in the HR above 45% of the HRR (Garber et al., 2011). Before the main experimental session, the participants completed an incremental test to volitional exhaustion using a cycling ergometer in the upright position. In this test, pulmonary oxygen consumption ( $\dot{V}$ O_2_) was measured, and the value was used to evaluate the ExL and ExM intensities performed in the main study as the percentage of the peak $\dot{V}$ O_2_ ( $\dot{V}$ O_2peak_). TRS sensors were placed over the forehead to evaluate oxygenation in the PFC during the experimental sessions. During exercise, ratings of perceived exertion (RPE) were evaluated on a 6–20 Borg scale (Borg, 1970) at the end of the ExL and ExM periods. Participants’ moods were evaluated before and after the cycling exercise using the Profile of Moods Scale-Brief (POMS-B) (McNair DM, 2007).

### 2.3 Experimental procedures

#### 2.3.1 Cycling ergometer incremental test

Before the main session of the NIRS study, each participant completed an incremental exercise test to volitional exhaustion using a cycling ergometer (Torqualizer ef med 600, h/p/cosmos, Nussdorf, Germany) in the upright position, starting at a WR of 20 W, with a subsequent increase in the WR at 20 W/min or 25 W/min according to the participant’s fitness level. Prior to the start of the test, the participants were instrumented and then they sat quietly for 2 min on the cycling ergometer. Respiratory variables were measured using an online gas analyzer (COSMED, Italy) in breath-by-breath mode, and $\dot{V}$ O_2_ and CO_2_ ( $\dot{V}$ CO_2_) production, minute ventilation ( $\dot{V}$ _E_), breathing frequency (R_f_), end-tidal CO_2_ (P_*ET*_CO_2_), and the respiratory exchange ratio (RER) were continuously measured. All data were averaged at 15-s intervals. The participants were instructed to pedal at a cadence of 60–70 rpm until volitional exhaustion or failure to maintain cadence of ≥55 rpm while they were verbally encouraged to reach maximum effort. The $\dot{V}$ O_2peak_ was confirmed if the following criteria were met: 1) measured HR_max_ ≥ age-predicted HR_max_ (220 age) minus 10 beats/min, 2) $\dot{V}$ O_2_ increased <150 mL/min, and 3) RER was ≥1.10. The $\dot{V}$ O_2peak_ was defined as the maximum 15-s average $\dot{V}$ O_2_ at maximum effort.

#### 2.3.2 Cardiovascular and respiratory measurements

The HR was measured using a Polar WearLink heart rate sensor with Bluetooth (Polar Electro Oy, Kempele, Finland). The systolic and diastolic BPs (SBP and DBP, respectively) were measured using an automated upper-arm cuff-oscillometric sphygmomanometer (Omron HEM-7120; Kyoto, Japan). BP was assessed at the baseline (rest), 3 min (Ex3), 13 min (Ex13), and 20 min (Ex20) after exercise initiation, and 5 min (Post5), 10 min (Post10), and 15 min (Post15) after exercise termination. Respiratory gas exchange variables were measured at rest, Ex3, Ex13, at the end of ExL (Ex17), Ex20, Post5, Post10, and Post15 time points, in breath-by-breath mode, using an online gas analyzer (Quark CPET, Cosmed) as described previously. All data were averaged over the last 15 s of the rest, Ex3, Ex13, Ex17, Ex20, Post5, Post10, and Post15 periods. During the exercise sessions, measurements were started 2 min before exercise initiation and continued for 15 min after the exercises were completed.

#### 2.3.3 Mood measurements

Participants’ moods before and after the cycling protocol were evaluated using the POMS-B. The POMS-B rates self-reported the mood across six dimensions: tension/anxiety (tension), depression/dejection (depression), anger/hostility (anger), fatigue/inertia (fatigue), confusion/bewilderment (confusion), and vigor/activity (vigor) (McNair DM, 2007).

#### 2.3.4 NIRS measurements

A portable three-wavelength TRS system (tNIRS-1; Hamamatsu Photonics K.K., Hamamatsu, Japan) was used for quantitative measurements of oxygenation in the PFC. The TRS system applies a time-correlated single-photon counting method. The precise methodology is described in detail in previous studies (Ijichi et al., 2005; Torricelli et al., 2014; Lange and Tachtsidis, 2019). Briefly, the system consists of 3-picosecond (ps) light pulses with different wavelengths (755, 816, and 850 nm), a 100-ps duration at a repetition frequency of 5 MHz as the pulse light source, a photon-counting head for single-photon detection, and signal-processing circuits for time-resolved measurements. By acquiring the distribution of the times of flight of photons propagating from the source to the detector, it is possible to enhance depth discrimination of absorption changes, thereby enabling the rejection of extra-cerebral effects (Wabnitz et al., 2020). The light emission and detection optodes were positioned on the forehead just below the hairline at a 40-mm inter-optode distance. With this optode spacing, the TRS can monitor hemodynamic responses around the gray matter region because the photons passing through the head convey the intracerebral hemodynamic responses (Ohmae et al., 2006). Based on our previous study (Hiura et al., 2018b), the position of TRS probes was based on the International EEG 10–20 system for electrode placement (Klem et al., 1999). The optodes were placed over the left and right forehead between Fp1 and F3 and between Fp2 and Fp4 to maximize the probability of photon transmission through the lateral portions of Brodmann areas 9 and 46 (Okamoto et al., 2004). According to the computed tomography scan of a representative participant, the explored volume included the dorsolateral PFC (DLPFC) in the middle frontal gyrus. The optodes were fixed using black rubber to prevent stray light from reaching the detector. The covered optodes were firmly affixed to the skin using transparent tape. Furthermore, to prevent movement, a cap made of opaque cloth was placed over the sensors. The photons can pass through the scalp, skull, and frontal lobe to a depth of several centimeters, with only minimal influence from skin blood flow (Fantini, 2014; Lange and Tachtsidis, 2019). As TRS provides absolute values of [HbO] and [HbR], [HbT] is determined as the sum of [HbO] and [HbR], with ScO_2_ and CBV calculated as follows:

$$ScO_{2}\;\left(\%\right)=\frac{\left[HbO\right]}{\left[HbO\right]+\left[HbR\right]},$$ (1)

$$CBV\;\left(mL/100\;g\right)=\frac{HbT\times {MW}_{Hb}}{HGB\times D_{bt}},$$ (2)

where the square brackets indicate the respective hemoglobin concentrations (μM) obtained by TRS, MW_Hb_ is the molecular weight of hemoglobin (64,500), HGB is the blood hemoglobin concentration (g/dL), and D_bt_ is the brain tissue density (1.05 g/mL) (Ijichi et al., 2005; Roche-Labarbe et al., 2010).

#### 2.3.5 Estimation of CMRO_2_

Based on the Fick principle, CMRO_2_ was calculated as follows:

$${CMRO}_{2}=k\times CBF\times HGB\times OEF,$$ (3)

where k = 1.39 [ml (O_2_)/g (HGB)] is the amount of O_2_ bound to HGB at complete saturation and HGB is the blood hemoglobin concentration (g/dL). As OEF equals to the arteriovenous oxygen difference, Eq. 3 can be rewritten as follows:

$${CMRO}_{2}=1.39\times CBF\times HGB\times \left({SaO}_{2}-SvO_{2}\right).$$ (4)

ScO_2_ was estimated using the following equation (Watzman et al., 2000):

$$ScO_{2}=\alpha SaO_{2}+\beta SvO_{2},with\;\alpha +\beta =1,$$ (5)

where α and β are constants, and α can be assumed as 0.25.

Using these formulas and applying SaO_2_ and ScO_2_, we obtained the following equation:

$$CMRO_{2}=CBF\times 1.39\times HGB\times \frac{4}{3}\left({SaO}_{2}-ScO_{2}\right).$$ (6)

If oxygenation, represented by ScO_2_, is measured simultaneously with CBF, CMRO_2_ is obtained. However, CBF could not be obtained by TRS for calculating CMRO_2_ combined with individual ScO_2_. Consequently, CBF was estimated using CBV, assuming a constant power law relationship between changes in CBF and CBV (Ito et al., 2001; Ito et al., 2003). Since we cannot clarify the ratio of the contribution from the arterial and venous components during exercise and HGB was not obtained in the present study, the ratio of CMRO_2_ to CMRO_2_ at rest (rCMRO_2_) was considered referring to previous studies using NIRS (Roche-Labarbe et al., 2010). rCMRO_2_ was calculated as follows:

$$rCMRO_{2}=\frac{CMRO_{2}}{CMRO_{2\;rest}}=\frac{HGB}{H{GB}_{rest}}\times {\left(\frac{CBV}{{CBV}_{rest}}\right)}^{\gamma }\times \left(\frac{SaO_{2}-ScO_{2}}{SaO_{2\;rest}-ScO_{2rest}}\right),$$ (7)

with γ = 3.4, induced by a previous study which demonstrated CBV = 1.09 × CBF^0.29^ (Ito et al., 2003), and the subscript “rest” indicates baseline values.

Additionally, the ratio of CBF to CBF at rest (rCBF) and the ratio of OEF to OEF at rest (rOEF) are as follows:

$$rCBF={\left(\frac{CBV}{{CBV}_{rest}}\right)}^{\gamma },$$ (8)

$$rOEF=\frac{SaO_{2}-ScO_{2}}{SaO_{2\;rest}-ScO_{2rest}}.$$ (9)

As the HGB values for six participants were examined within a month of the main study, we referred to these values only to clarify whether [HbT] was an outlier. Referring to Eq. 7, individual HGB was not necessary to calculate rCMRO_2_ and relative changes in CBV, i.e., the ratio of CBV to CBV_rest_ (rCBV). We calculated rCMRO_2_ when ScO_2_ or [HbT] changed significantly from the baseline. As arterial and venous blood samples were not available in the present study, we assumed that HGB and SaO_2_ would not change during ExL and ExM or after completion of the exercise protocol. We assumed SaO_2_ to be 98% throughout the exercise protocol.

As the TRS system (tNIRS-1) sampled data at 5-s intervals, all data were averaged over the last 15 s of the rest, Ex3, Ex13, Ex17, Ex20, Post5, Post10, and Post15 time points to compare NIRS data with respiratory variables in the same time window. To obtain the best possible baseline and postexercise conditions, NIRS signals were observed as a fraction of fluctuations in HR, P_*ET*_CO_2_, and $\dot{V}$ O_2_ so that physiological confounders, including artifacts caused by body movement, were excluded. During the exercise protocol, measurements were started 2 min before exercise initiation and continued for 15 min after the exercises were completed. To evaluate the time course of variables, bins of 15-s data were sampled every 1 min. For the baseline data, we applied averaged data of 30 s, avoiding a duration of 30 s immediately before the start of exercise as NIRS signals in the PFC increase before the initiation of voluntary leg cycling (Matsukawa et al., 2015; Asahara et al., 2022) according to the central command (Nowak et al., 1999).

### 2.4 Statistical analysis

Data were analyzed using GraphPad Prism 9 (San Diego, CA, United States) and SPSS version 25 (IBM, Armonk, NY, United States). Power calculation using G*Power showed that, setting the effect size as 0.40 with the number of measurements as 3 (rest, ExL, and ExM), a sample size of n = 6 for each level is sufficient (alpha level<0.05 and statistical power >90%) (Hiura et al., 2022). A previous study using the TRS system (tNIRS-1) explored changes in [HbT] during a 13-min session, which consisted of 70% and 80% of $\dot{V}$ O_2peak_ on a rowing ergometer (n = 11), and demonstrated an effect size of 0.881. Accordingly, assuming the effect size as 0.40 in the current study, we considered a sample size of n = 6 in each level for one-way repeated-measures analysis of variance (ANOVA) as appropriate. However, consistent with the previous PET study, we investigated 12 participants. The average data are expressed as the arithmetic mean ± standard deviation, unless stated otherwise. The normality of the distribution of data was evaluated using the Shapiro–Wilk test. One-way repeated-measures ANOVA with Tukey’s honestly significant difference (HSD) *post-hoc* test was performed to evaluate the differences measured in the rest period and during and after the exercise protocol. Perceived exertion and mood status were evaluated between conditions, at the baseline and postexercise, using either paired Student’s t-test or the Wilcoxon matched-pairs signed rank test, as appropriate per the data distribution. The magnitude of the difference in NIRS variables was assessed using effect sizes (partial eta squared; η^2^) defined as follows: small (≥0.01 to <0.06), medium (≥0.06 to <0.14), and large (≥0.14) for η^2^ (Cohen, 1988). To compare the changes in NIRS variables between the left and right PFC, and the changes in rCMRO_2_ and rCBV across time points, we used two-way (group-by-time) repeated-measures ANOVA with Sidak’s multiple comparison test. A *p*-value of <0.05 was considered significant.

## 3 Results

### 3.1 Cardiovascular variables

Table 1 summarizes the cardiovascular variables during the exercise protocol. All cardiovascular variables increased during exercise and were higher at Ex20 than Ex13. After exercise termination, the HR remained slightly higher than that at rest, except at Post15. Compared with the resting baseline, SBP decreased at Post10 and Post15 and MBP decreased at Post15, while DBP remained at baseline levels after exercise termination. HR reached 30% ± 2% HRR at Ex13% and 46% ± 3% HRR at Ex20.

**TABLE 1:** Cardiovascular variables in response to the 17-min low-intensity constant-load cycling exercise, followed by the 3-min moderate-intensity constant-load cycling exercise.

	Rest	Ex3	Ex13	Ex20	Post5	Post10	Post15
Heart rate (beats per min^-1^)	62 ± 6	100 ± 5***	103 ± 6***	120 ± 5***, §§	69 ± 8**	66 ± 7*	65 ± 7
SBP (mmHg)	126 ± 10	149 ± 13***	150 ± 10***	160 ± 13***, §§	129 ± 10	122 ± 9*	119 ± 7*
DBP (mmHg)	74 ± 8	84 ± 9***	81 ± 7	87 ± 8**, §§	75 ± 8	73 ± 9	72 ± 9
MBP (mmHg)	91 ± 7	106 ± 10***	104 ± 7***	111 ± 9***, §§	93 ± 6	89 ± 7	88 ± 6*

Values are expressed as the mean ± standard deviation (n = 12) at the baseline (rest), 3 min (Ex3), 13 min (Ex13), and 20 min (Ex20) min after exercise initiation, and 5 min (Post5), 10 min (Post10), and 15 min (Post15) min after exercise termination.

SBP: systolic blood pressure; DBP: diastolic blood pressure; MBP: mean blood pressure.

Significant difference from rest: *p < 0.05, **p < 0.01, and ***p < 0.001. Significant difference from Ex13: §§*p* < 0.01.

### 3.2 Respiratory variables

The respiratory variables during the exercise protocol are summarized in Table 2. All respiratory variables, except the RER, increased during exercise and reached a steady state during ExL, returning to the baseline level after exercise termination. After the exercise intensity was changed, $\dot{V}$ _E_, $\dot{V}$ O_2_, $\dot{V}$ CO_2_, and P_*ET*_CO_2_ increased at Ex20 compared with Ex17. Based on the values of $\dot{V}$ O_2peak_ obtained during the incremental exercise test to volitional exhaustion in the upright position, $\dot{V}$ O_2_ reached 33% ± 5% and 46% ± 4% $\dot{V}$ O_2peak_ at ExL and ExM, respectively.

**TABLE 2:** Respiratory variables in response to the 20-min cycling exercise comprising 17-min low-intensity constant-load cycling exercise followed by the 3-min moderate-intensity constant-load cycling exercise, obtained in the near-infrared spectroscopy study.

	Rest	Ex3	Ex13	Ex17	Ex20	Post5	Post10	Post15
$\dot{V}$ _E_ (L min^-1^)	11 ± 2	26 ± 5***	31 ± 5***	31 ± 4***	40 ± 6***, §§	11 ± 3	10 ± 2	9 ± 3
R_f_ (breaths/min)	18 ± 4	24 ± 6**	28 ± 6***	28 ± 6***	28 ± 6***	18 ± 5	17 ± 5	15 ± 5
$\dot{V}$ O_2_ (L min^-1^)	0.4 ± 0.1	1.1 ± 0.1***	1.2 ± 0.1***	1.2 ± 0.1***	1.6 ± 0.2***, §§	0.3 ± 0.1	0.3 ± 0.1	0.3 ± 0.1
Relative $\dot{V}$ _O2_ (mL min^-1^ kg^-1^)	5.6 ± 1.3	15.4 ± 2.0***	16.7 ± 2.1***	17.4 ± 3.0***	23.1 ± 3.6***, §§	4.6 ± 0.9	4.5 ± 1.2	4.1 ± 1.0
$\dot{V}$ CO_2_ (L min^-1^)	0.3 ± 0.1	1.0 ± 0.1***	1.1 ± 0.1***	1.1 ± 0.2***	1.6 ± 0.2***, §§	0.3 ± 0.1	0.3 ± 0.1	0.3 ± 0.1
RER	0.88 ± 0.04	0.89 ± 0.07	0.94 ± 0.04*	0.94 ± 0.04*	0.98 ± 0.07*	1.03 ± 0.11*	0.89 ± 0.09	0.89 ± 0.09
P_*ET*_CO_2_ (mmHg)	36.8 ± 3.3	44.7 ± 4.9**	43.5 ± 3.7**	43.7 ± 3.7**	46.1 ± 4.5***, §	35.3 ± 2.8	34.8 ± 4.2	34.6 ± 4.8

Values are expressed as the mean ± standard deviation (n = 12) at the baseline (rest), 3 min (Ex3), 13 min (Ex13), 17 min (Ex17), and 20 min (Ex20) min after exercise initiation, and 5 min (Post5), 10 min (Post10), and 15 min (Post15) min after exercise termination.

$\dot{V}$ _E_: minute ventilation; R_f_: breathing frequency; $\dot{V}$ O_2_: pulmonary oxygen consumption; $\dot{V}$ CO_2_: CO_2_ production; RER: respiratory exchange ratio; P_ET_CO_2_: end-tidal carbon dioxide.

Significant difference from rest: *p < 0.05, **p < 0.01, and ***p < 0.001. Significant difference from Ex13: §*p* < 0.01.

### 3.3 Perceived exertion and mood status

During exercise, RPE changed from 8.8 ± 1.1 a.u. (arbitrary units) for ExL to 11.4 ± 1.68 a.u. for ExM (*p* < 0.0001). The mood ratings for the six dimensions of the POMS-B at rest and after exercise are summarized in Table 3. There was a significant decrease in mood ratings for fatigue (*p* = 0.03), with a tendency for a decrease in tension (*p* = 0.250), anger (*p* = 0.187), and confusion (*p* = 0.121).

**TABLE 3:** Descriptive statistics for the subjective mood ratings collected before and after exercise.

Factors of POMS-B	Tension	Depression	Anger	Vigor	Fatigue	Confusion
Baseline	2.5 ± 2.8	0.7 ± 1.2	1.3 ± 2.0	8.3 ± 1.2	5.2 ± 4.7	4.7 ± 2.8
Postexercise	1.5 ± 2.6	0.4 ± 0.9	0.3 ± 0.9	8.8 ± 4.7	2.5 ± 2.8*	3.5 ± 2.4

Values are shown as the mean ± standard deviation (a.u.).

Significant difference from the baseline: *p < 0.05; POMS-B: Profile of Moods Scale-Brief.

### 3.4 NIRS variables

**FIGURE 2:**
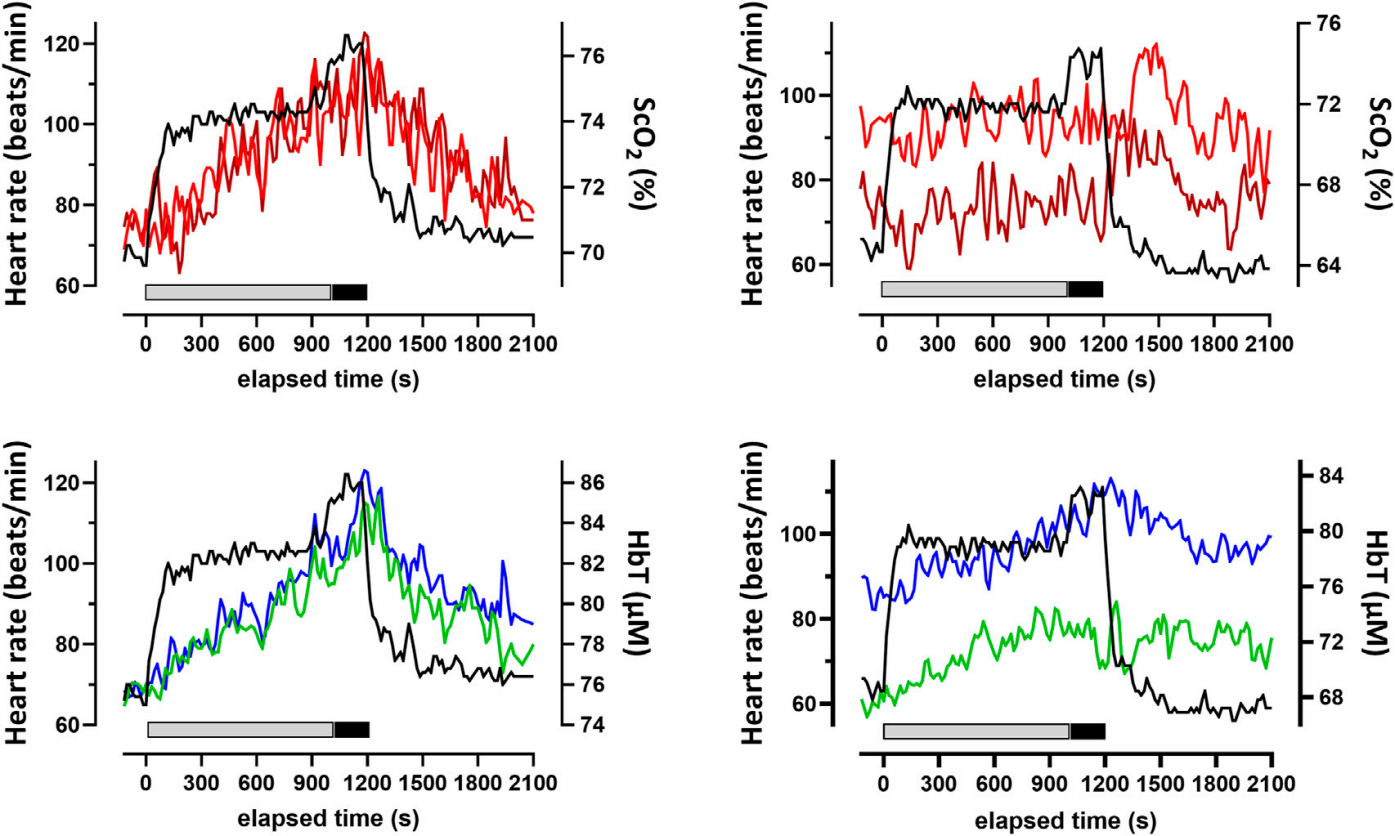
Typical changes for two participants (left and right) in the heart rate and cerebral oxygenation variables obtained by NIRS for the prefrontal cortex during and after the 20-min constant-load cycling protocol, comprising 17 min of low-intensity and 3 min of moderate-intensity exercise. Data were averaged over 15 s at each time point. Changes in ScO_2_ for the left (red) and right (brown) PFC and HbT for the left (green) and right (blue) prefrontal cortex are depicted. The thick bars below the NIRS parameter changes indicate the periods of the exercise session of low (gray) and moderate intensity (black). ScO_2_: cerebral hemoglobin oxygen saturation; HbT: total hemoglobin concentration in the brain tissue; NIRS: near-infrared spectroscopy.

**FIGURE 3:**
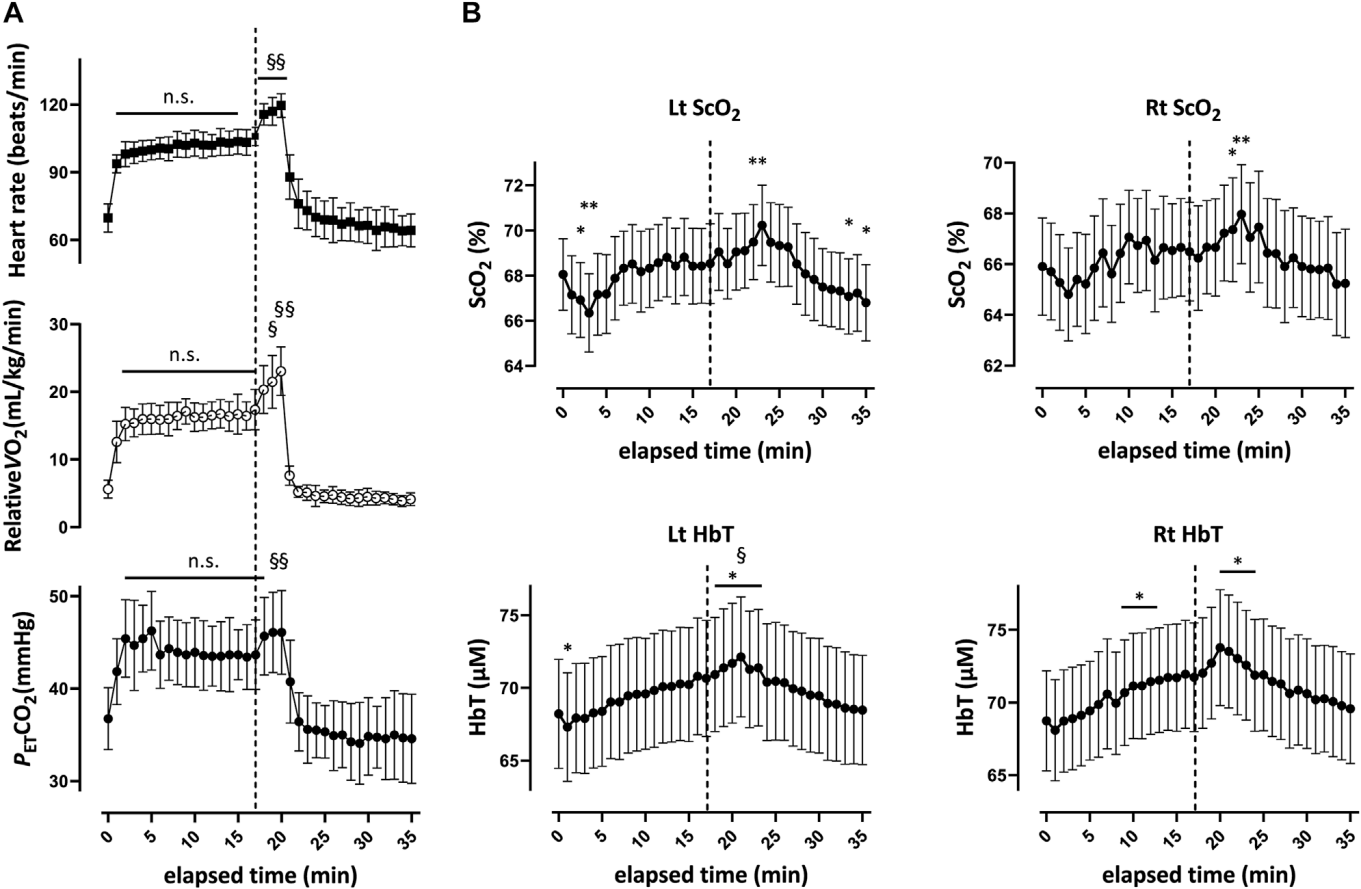
Respiratory variables **(A)** and cerebral oxygenation variables obtained by near-infrared spectroscopy for the prefrontal cortex **(B)** during and after the 20-min constant-load cycling protocol, comprising 17 min of low-intensity and 3 min of moderate-intensity exercise. Data were averaged over 15 s at each time point. $\dot{V}$ O_2_: pulmonary oxygen consumption; P_ET_CO_2_: end-tidal carbon dioxide; ScO_2_: cerebral hemoglobin oxygen saturation; HbT: total hemoglobin concentration in the brain tissue; Lt: left; Rt: right. Significant difference compared to the baseline: *p < 0.05. Significant difference compared to the end of light-intensity exercise (17 min); §*p* < 0.05. Statistical differences between each time point evaluated using one-way analysis of variance for repeated measures and Turkey’s *post hoc* multiple comparison test.

**FIGURE 4:**
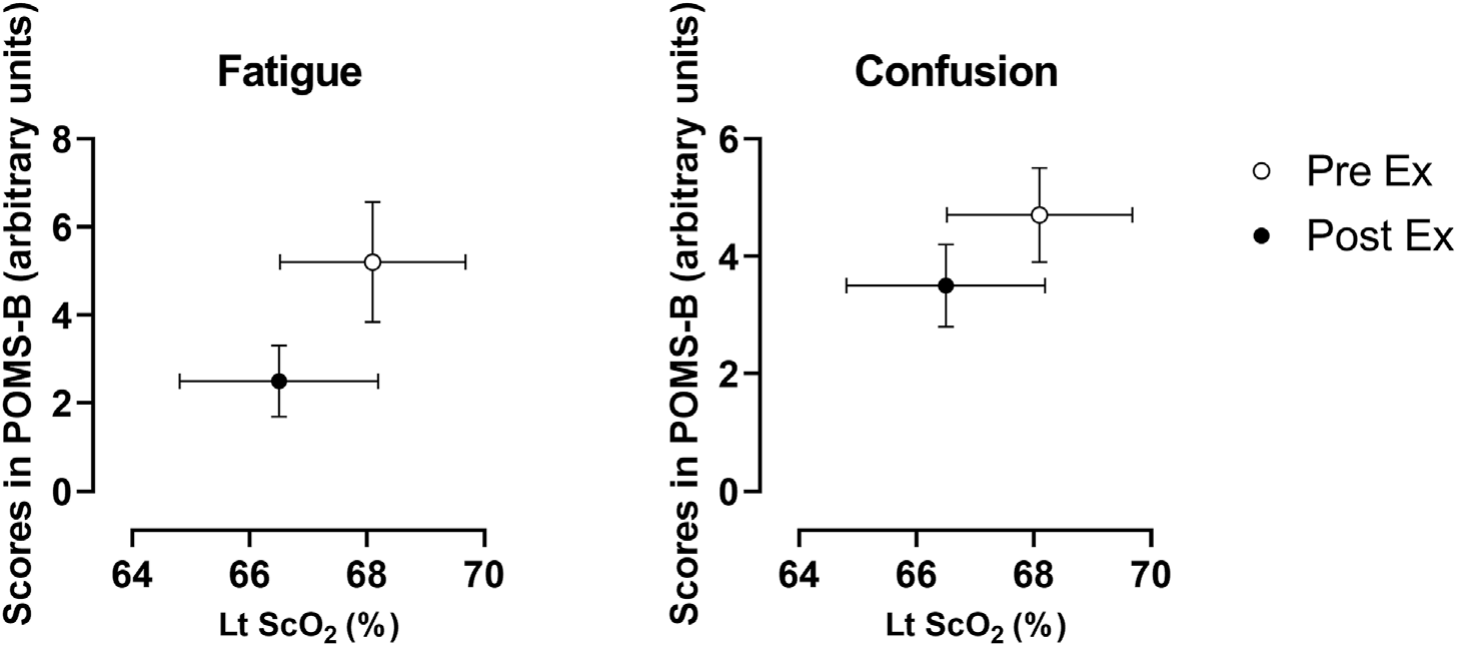
Changes in mood ratings for fatigue and confusion and ScO_2_ for the left prefrontal cortex. Data were derived from all of the participants (n = 12) and expressed as the mean ± standard error of the mean. Lt: left; PreEx: before the exercise started; PostEx: after the exercise terminated.

Two typical examples of changes in ScO_2_ and [HbT] are shown in conjunction with the HR in Figure 2. Changes in these NIRS and physiological variables for all participants are shown in Supplementary Figure S1. Changes in ScO_2_ and [HbT] throughout the exercise protocol are presented, along with changes in HR, P_ET_CO_2_, and relative $\dot{V}$ O_2_, in Figure 3. Regarding the changing patterns of ScO_2_ and [HbT], differences between the left and right PFC were evaluated. Interaction effects were identified neither for ScO_2_ [F (35, 770) = 0.7024; *p* = 0.9021] nor for [HbT] [F (35, 770) = 0.2155; *p* > 0.999]. There was a main effect of the side factor for ScO_2_ [F (1, 11) = 8.982; *p* = 0.012] and [HbT] [F (1, 11) = 5.154; *p* = 0.044], with ScO_2_ being higher on the left side than on the right side and [HbT] being higher on the right side than on the left side for most of the experimental protocol period. ScO_2_ decreased to its nadir at 3 min after exercise initiation, with a significant decrease in the left PFC, and then returned to the baseline until the end of the ExM period. At 2 min and 3 min after exercise termination, ScO_2_ was higher than that at rest. In the left PFC, ScO_2_ at 13 min and 15 min after exercise termination was lower than that at rest. [HbT] continued to increase toward the end of ExM, except at 1 min after exercise initiation in the left PFC, and was higher from the end of ExM to 3 min after exercise termination than that at rest on both sides of the PFC. [HbT] returned to the baseline from 4 min after exercise termination and remained at this level thereafter. A summary of the NIRS variables during the exercise protocol is shown in Table 4. In the left PFC, ScO_2_ decreased at Ex3 and Post15 compared with that at rest, while ScO_2_ did not change throughout the course in the right PFC. [HbT] gradually increased in both sides of the PFC during exercise and was significantly higher at ExM than at rest. The magnitude of change in [HbT] was larger than that in the other variables of oxygenation. [HbO] increased at Ex20 in both sides of the PFC, and [HbR] did not change significantly during the session, except for HbR at Ex3 in the left PFC. As mood ratings for fatigue significantly decreased and confusion tended to decreased after exercise termination, ScO_2_ decreased significantly at Post15 compared with rest (Figure 4).

**TABLE 4:** Changes in NIRS signals in response to constant-load cycling exercise protocol, 17-min of low-intensity exercise, followed by the 3-min moderate-intensity exercise.

	Rest	Ex3	Ex13	Ex17	Ex20	Post5	Post10	Post15	ES (η^2^-partial)
ScO_2_ (%)									
Rt	65.4 ± 6.4	64.8 ± 6.3	66.2 ± 7.0	66.5 ± 6.7	66.7 ± 6.6	67.5 ± 7.6*	65.9 ± 6.9	65.0 ± 7.3	0.296
Lt	68.1 ± 5.5	66.5 ± 6.0*	68.3 ± 5.8	68.4 ± 6.1	68.8 ± 5.8	68.7 ± 6.3	67.3 ± 5.9	66.5 ± 5.9*, §	0.438
HbT (μM)									
Rt	68.7 ± 11.9	68.9 ± 12.2	71.5 ± 12.5*	72.0 ± 13.0*	73.8 ± 13.8*, §	71.9 ± 13.3*	70.6 ± 13.1	69.6 ± 13.0	0.482
Lt	68.2 ± 13.0	67.9 ± 13.1	70.1 ± 13.2*	70.7 ± 13.8*	71.7 ± 14.3*	70.5 ± 14.0*	69.5 ± 13.6	68.3 ± 13.2	0.524
HbO (μM)									
Rt	45.5 ± 9.8	44.8 ± 9.3	47.5 ± 10.3	47.9 ± 10.8	49.4 ± 11.3*	48.7 ± 11.5*	46.7 ± 10.6	45.6 ± 10.8	0.415
Lt	46.4 ± 10.2	45.3 ± 10.3	48.1 ± 10.8	48.6 ± 11.4*	49.6 ± 11.9*	48.7 ± 11.7*	47.0 ± 10.9	45.7 ± 10.5§	0.494
HbR (μM)									
Rt	23.5 ± 5.3	24.2 ± 5.4	24.0 ± 5.5	23.8 ± 5.3	24.4 ± 5.7	23.1 ± 5.5	23.9 ± 5.5	23.9 ± 5.6	0.182
Lt	21.8 ± 4.8	22.6 ± 5.1*	22.0 ± 4.6	22.1 ± 4.9	22.1 ± 4.8	21.8 ± 5.0	22.5 ± 4.8	22.5 ± 5.0	0.149

Values are expressed as the mean ± standard deviation (n = 12) at the baseline (rest), 3 min (Ex3), 13 min (Ex13), 17 min (Ex17), and 20 min (Ex20) min after exercise initiation, and 5 min (Post5), 10 min (Post10), and 15 min (Post15) after exercise termination.

The data were averaged over 15 s at each time point.

ScO_2_: cerebral hemoglobin oxygen saturation; HbT: total hemoglobin concentration in the brain tissue; HbO: oxyhemoglobin concentration in the brain tissue; HbR: deoxyhemoglobin concentration in the brain tissue; Rt: right; Lt: left.

Significant difference from the rest: *p < 0.05 and **p < 0.01. Significant difference from Ex17: §*p* < 0.05.

ES: effect size.

### 3.5 Relationships among rCMRO_2_, rCBV, rCBF, and rOEF

**FIGURE 5:**
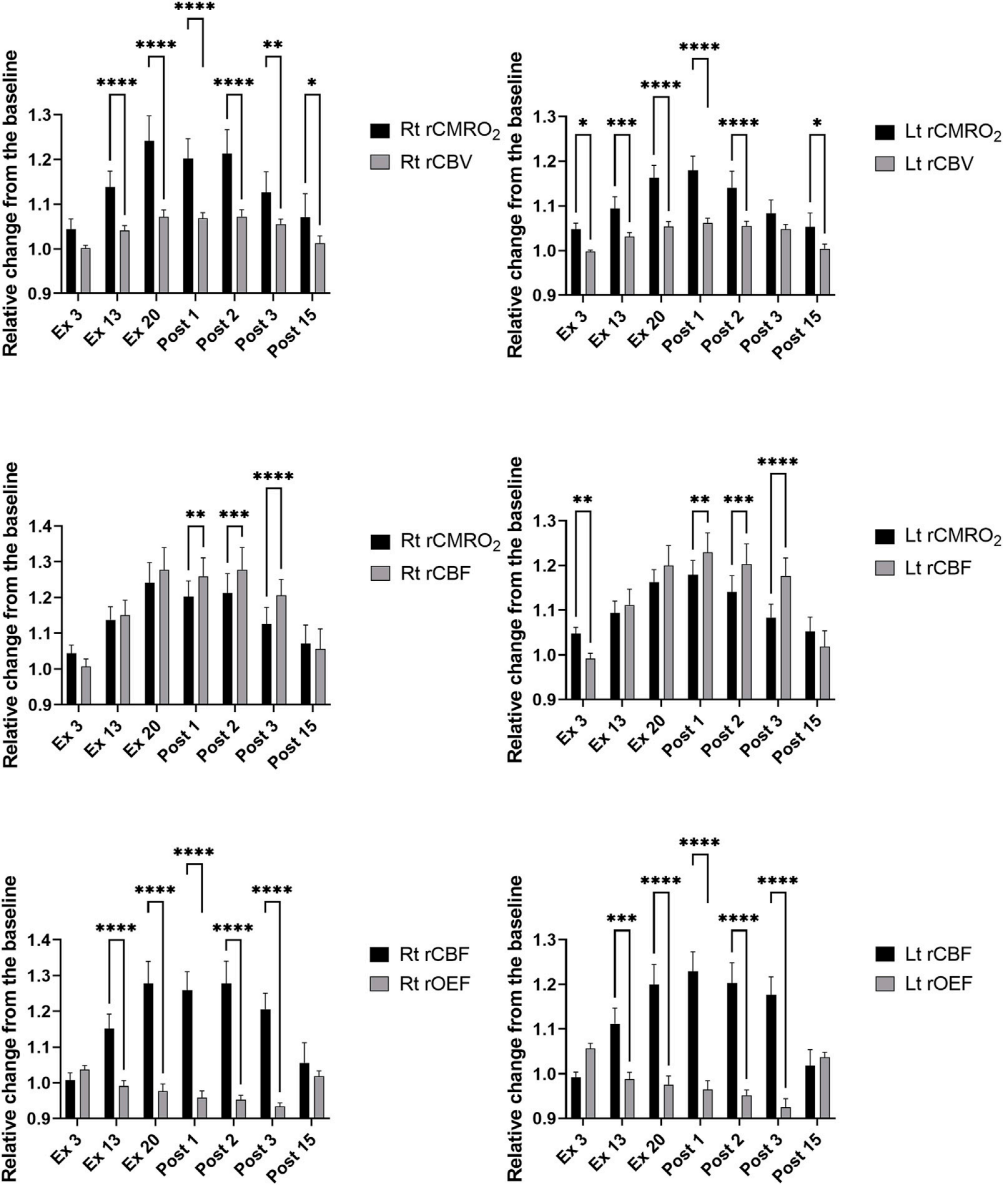
Relative changes from the baseline in the right and left prefrontal in the cerebral metabolic rate for oxygen and cerebral blood volume (upper), in the cerebral metabolic rate for oxygen and cerebral blood flow (middle), and in the cerebral blood flow and oxygen extraction fraction (lower) during and after the 20-min constant-load cycling protocol, comprising 17 min of low-intensity and 3 min of moderate-intensity exercise. Data were averaged over 15s at each time point and expressed as the mean ± standard error of the mean. rCMRO_2_: relative changes from the baseline in the cerebral metabolic rate for oxygen; rCBV: relative changes from the baseline in the cerebral blood volume; rCBF: the relative changes from the baseline in cerebral blood flow; rOEF: the relative changes from the baseline in the oxygen extract fraction; Lt: left; Rt: right; rest: baseline; Ex3, Ex13, and Ex20: 3, 13, and 20 min after exercise initiation, respectively; Post1, Post2, Post3, and Post15: 1, 2, 3, and 15 min after exercise termination, respectively, difference between rCMRO_2_ and rCBV, rCMRO_2_ and rCBF, and rCBF and rOEF at each time point; **p < 0.05, ***p < 0.001, and ****p < 0.0001; Sidak’s multiple comparison test.

Based on the changes in ScO_2_ and [HbT] (Figure 3B), we calculated rCMRO_2_, rCBV, rCBF, and rOEF using Eqs 7–9. Comparisons between rCMRO_2_ and rCBV, rCMRO_2_ and rCBF, and rCBF and rOEF obtained at each time point for both sides of the PFC are shown in Figure 5. We selected the time points where either ScO_2_ or HbT changed significantly on either side of the PFC compared with that at rest. The relationships between rCMRO_2_ and rCBF, rCMRO2 and rCBF, and rCBF and rOEF differed over the time course of the exercise protocol for the right and left PFC [F (6, 66) = 6.175 and 4.335, *p* < 0.001 and *p* = 0.001; F (6, 66) = 7.469 and 14.32, *p* < 0.0001 and *p* = 0.0001; and F (6, 66) = 19.89 and 24.6, *p* < 0.0001 and *p* = 0.0001, respectively]. A summary of rCMRO_2_, rCBV, rCBF, and rOEF during the exercise protocol is shown in Table 5. During ExL, rCMRO_2_ and rCBV did not change except for at Ex13 in the right PFC. CMRO_2_ surpassed rCBV at Ex3, and this relationship remained until immediately after exercise termination. CMRO_2_ surpassed rCBF at Ex3 in the left PFC; however, this relationship changed to the opposite direction in that CBF surpassed rCMRO_2_. At Ex20 and immediately after exercise termination, CMRO_2_, rCBV, and rCBF were increased compared with that at Ex3 on both sides of the PFC. Compared with Ex13, rCMRO_2_, rCBV, and rCBF were higher at Ex20 and Post1 on the right of the PFC. rOEF decreased at Ex20 and immediately after exercise termination compared with Ex3. The magnitude of change in rCBF was larger than that in rCMRO_2_, rCBV, and rOEF.

**TABLE 5:** Changes in the relative changes from the baseline cerebral metabolic rate for oxygen and cerebral blood volume in response to 17-min low-intensity cycling ergometer exercises, followed by 3-min moderate-intensity cycling ergometer exercises.

	Ex3	Ex13	Ex20	Post1	Post2	Post3	Post15	ES (η^2^-partial)
rCMRO_2_								
Rt	1.04 ± 0.08	1.14 ± 0.13	1.24 ± 0.20**, §	1.20 ± 0.15**	1.21 ± 0.19**	1.13 ± 0.16	1.07 ± 0.18	0.466
Lt	1.05 ± 0.05	1.09 ± 0.09	1.16 ± 0.10**	1.18 ± 0.11**, §	1.14 ± 0.13*	1.08 ± 0.11	1.05 ± 0.11	0.406
rCBV								
Rt	1.00 ± 0.02	1.04 ± 0.04**	1.07 ± 0.05**, §	1.07 ± 0.04**	1.07 ± 0.05**, §	1.05 ± 0.04**	1.01 ± 0.05	0.633
Lt	1.00 ± 0.02	1.03 ± 0.04	1.06 ± 0.05**	1.07 ± 0.05**, §	1.06 ± 0.05*	1.05 ± 0.04	1.01 ± 0.04	0.406
rCBF								
Rt	1.01 ± 0.07	1.15 ± 0.14**	1.28 ± 0.22**, §	1.26 ± 0.18**	1.28 ± 0.22**, §	1.21 ± 0.16**	1.06 ± 0.20	0.631
Lt	0.99 ± 0.04	1.11 ± 0.12**	1.20 ± 0.15**	1.23 ± 0.15**, §	1.20 ± 0.16**, §	1.18 ± 0.14**	1.02 ± 0.13, §	0.641
rOEF								
Rt	1.04 ± 0.04	0.99 ± 0.05	0.98 ± 0.07*, §	0.96 ± 0.06**	0.95 ± 0.05**	0.93 ± 0.04**, §	1.02 ± 0.05	0.456
Lt	1.06 ± 0.04	0.99 ± 0.05**	0.98 ± 0.07**, §	0.97 ± 0.07**	0.95 ± 0.04**	0.93 ± 0.07**, §	1.04 ± 0.04, §	0.608

Values are expressed as the mean ± standard deviation (n = 12) at 3 min (Ex3), 13 min (Ex13), and 20 min (Ex20) after exercise initiation and 1 min (Post1), 2 min (Post2), 3 min (Post3), and 15 min (Post15) after exercise termination.

The data were averaged over 15 s for each time point.

rCMRO_2_: relative changes from the baseline in the cerebral metabolic rate for oxygen; rCBV: the relative changes from the baseline in the cerebral blood volume; rCBF: the relative changes from the baseline in cerebral blood flow; rOEF: the relative changes from the baseline in oxygen extract fraction; Rt: right; Lt: left.

Significance of difference from Ex 3: *p < 0.05 and **p < 0.001. Significance of difference from Ex13: §*p* < 0.05.

ES: effect size.

rCMRO_2_, rCBV, rCBF, and rOEF are demonstrated when ScO_2_ or [HbT] changed significantly from the baseline.

## 4 Discussion

In this study, cerebral oxygen metabolism and perfusion during low- and moderate-intensity cycling exercises were examined by investigating ScO_2_ and [HbT]. While CBV can be calculated using ScO_2_ and HGB, we estimated rCBF assuming a constant power law relationship between CBV and CBF. There were several main findings of our study that estimated changes in CMRO_2_ and CBV using NIRS variables. First, rCBV increased significantly during ExL only in the right PFC, and ScO_2_ decreased in the initial phase of exercise, especially in the left PFC, which led to the difference between rCMRO_2_ and rCBV. Second, assuming the power law relationship between CBV and CBF, rCBF increased significantly during ExL, causing the difference between rCMRO_2_ and rCBF, with rCBF surpassing rCMRO_2_, which was contrary to the relationships between rCMRO_2_ and rCBV. Third, elevated cardiovascular variables during ExM, in conjunction with the augmented sense of effort, caused differences between rCMRO_2_ and rCBV and between rCMRO_2_ and rCBF. Fourth, after exercise termination (Post15), rCMRO_2_ increased and was greater than rCBV, while no differences were identified between rCMRO_2_ and rCBF and between rCBF and rOEF associated with PEH and positive changes in the mood status as well. These results demonstrate the estimated relationship between cerebral oxygen metabolism and perfusion during exercise explored by TRS.

### 4.1 Cerebral oxygen metabolism and perfusion in response to low- and moderate-intensity exercise

As studies have reported changes in oxygenation under various situations in the PFC, including maximal incremental tests and constant workload tasks ranging from low to supramaximal intensities (De Wachter et al., 2021), the present study addressed the important aspect of the relationship between cerebral oxygen metabolism and perfusion. During ExL, ScO_2_ decreased at Ex3 in the left PFC. This finding is consistent with that of a previous study (Takehara et al., 2017) that measured changes in [HbO] and [HbR] during low- and moderate-intensity cycling exercise, but the absolute values of ScO_2_ were not measured. Decrease in ScO_2_ can be attributed to the increase in CMRO_2_, provided that CBF does not decrease. At Ex3, rCMRO_2_ surpassed rCBV and rCBF in the left PFC. As the exercise continued with ExL, rCBV increased only in the right PFC at Ex13, but rCBF increased in both the sides. This is not compatible with the result from a previous PET study (Hiura et al., 2018a), in which participants and exercise intensity were similar to those in the present study, which showed no change in CBF in the PFC during ExL. It would be attributable to the methodological limitation for PET measurements in which the coefficient of variation was approximately 20% for CBF in our previous study (unpublished data). At Ex13, rCMRO_2_ surpassed rCBV, while rCMRO_2_ was compatible to rCBF.

By the end of ExM, ScO_2_ and [HbT] increased significantly, along with a simultaneous increase in the cardiovascular and respiratory variables and perceived exertion. rCBF increased during exercise in association of the cerebrovascular reactivity to CO_2_, with P_*ET*_CO_2_ ranging from 36–44 mmHg. These changes in NIRS variables caused an increase in rCMRO_2_ in the PFC at ExM compared with that at ExL and induced an opposite relationship between rCMRO_2_ and rCBF compared with that at ExL such that rCBF surpassed rCMRO_2_ immediately after exercise termination. This finding is associated with that of a previous study which demonstrated that the relationship between rCBF and CMRO_2_ during prolonged continuous visual stimulation was different at 1 min and 25 min of the observation period (Mintun et al., 2002). The study demonstrated that the relative changes in CMRO_2_ were higher than those in CBF at 25 min from the initiation of the stimulation, and this relationship was reversed at 1 min from the initiation. Further studies are needed to elucidate the effect of both the intensity and duration of exercise on the relationship between CMRO_2_ and CBF. After ExM termination, a situation in which rCBF surpassed rCMRO_2_ coincided with the decrease in rOEF, which seems to control rCMRO_2_ to be at a constant level. This phenomenon is associated with a previous study that explored the relationships among CBF, CMRO_2_, and OEF for the global brain, in which the maximal exercise during dehydration induced a decrease in CBF but compensator increases in OEF maintained CMRO_2_ (Trangmar et al., 2014).

We observed changes in cerebral oxygenation in response to higher-intensity exercise. However, the observed intensities of 33% ± 5% and 46% ± 4% $\dot{V}$ O_2peak_ at ExL and ExM, respectively, would be an underestimation because these intensities were based on the $\dot{V}$ O_2peak_ attained in an upright position, while a supine position was used in the exercise protocol. Since the $\dot{V}$ O_2peak_ in an upright position is higher than that in the supine position in healthy subjects (Wan et al., 2021), with a difference of nearly 10% in the relative $\dot{V}$ O_2peak_ (Wehrle et al., 2021), exercise intensities were calculated as 37% ± 6% and 51% ± 5% $\dot{V}$ O_2peak_ at ExL and ExM, respectively, based on the $\dot{V}$ O_2peak_ attained by an incremental test performed in the supine position.

### 4.2 Relationship between cerebral oxidative metabolism and perfusion associated with the functional role of the PFC during exercise

During low-intensity cycling exercise, CBF was shown to increase significantly in the bilateral sensorimotor cortex for the legs and cerebellum, with a moderate increase in the insular cortex and no increase in the PFC (Hiura et al., 2014; Hiura et al., 2018a). While neuronal activations for leg movement and central command result in a significant increase in CBF in these regions, we speculate that the associated neuronal activation for initiating exercise would result in an increase in rCMRO_2_, but not in rCBV and rCBF, at Ex3. To the best of our knowledge, no study has reported an increase in CBF in the PFC using PET during exercise, although the DLPFC has reciprocal interconnections with the premotor areas, basal ganglia, and cerebellum, by which the lateral part of the PFC can control broad aspects of motor behavior (Seeley et al., 2007; Tanji and Hoshi, 2008). Matsukawa et al. (2015) reported a decrease in [HbO] with a slight change in [HbR] and a substantial decrease in ScO_2_, which is in line with our results, and suggested a possible contribution of feedback to the DLPFC stimulated by mechanical limb motion. We speculated that it was not the change in CBF but the increase in CMRO_2_ that indicated a possible association between the DLPFC and other cortical regions that have executive control and autonomic regulation.

Exercise is associated with a positive effect on feelings of vigor and fatigue (Dishman et al., 2010). In particular, the PFC is associated with various features of affective processing (Davidson, 2003) and mood status (Monroe et al., 2020). A recent study performed by using NIRS demonstrated that increased changes in [HbO] were identified in the DLPFC after 10 min of running at an intensity of 50% $\dot{V}$ O_2peak_, which coincided with improved performance in the executive function, using the color-word matching Stroop task, and positive changes in the mood state (Damrongthai et al., 2021). Our results are in line with those of this previous study, wherein exercises of identical intensities and different modes (cycling instead of running) evoked positive changes in the mood state. As ScO_2_ in the left PFC significantly decreased at Post15, the tendency of dissociated coupling of rCMRO_2_ and rCBF (*p* = 0.113) may be attributable to the functional role of the PFC causing a positive effect on mood status after exercise. Regarding performance in the executive function, Endo et al. reported that the total time period for the Stroop test was improved after 15 min of low-intensity exercise (Endo et al., 2013) and that the incremental rate of prefrontal oxygenation may decrease in the progression of aging based on changes in [HbO] in the PFC during the Stroop task (Endo et al., 2018). Considering the change in [HbO] in the PFC in these studies, our results may also be associated with executive function in the PFC related to exercise.

As PEH was induced with a small change of 7 mmHg in SBP at most, the relationship between cerebral oxygen and perfusion was dissociated as rCMRO_2_ surpassed rCBV at Post15 but not rCBF, while SBP and MBP decreased compared with the baseline. However, to clarify if this dissociated relationship between cerebral oxygen metabolism and perfusion in the PFC contributes to its mechanism, another exercise protocol that will evoke a more severe PEH is needed in the future.

With our exercise protocol, no interaction effects were identified regarding ScO_2_ or [HbT] and the PFC side, although absolute values of ScO_2_ were greater in the left PFC and those of [HbT] were greater in the right PFC. This finding is consistent with that of a recent study (Asahara and Matsukawa, 2023) that explored changes in [HbO] and ScO_2_ in the PFC during low- and moderate-intensity one-legged cycling exercise using t-NIRS. The authors demonstrated that no laterality-related differences were observed in the changes in cerebral oxygenation.

### 4.3 Significance and perspective of exploring the relationship between CMRO_2_ and CBF

We estimated the relationship between CMRO_2_ and CBF using TRS NIRS since previous studies using PET have demonstrated that changes in CBF are not uniquely coupled to changes in oxidative metabolism. The relationships between CMRO_2_ and CBF have been examined in humans during low-intensity somatosensory and visual stimulation and during motor activity such as moving fingers at different rates (Mintun et al., 2002; Vafaee and Gjedde, 2004; Vafaee et al., 2012). Spatially dissociated changes in CMRO_2_ and CBF in several cortical and subcortical regions of the brain during the right finger-to-thumb movement were suggested, demonstrating that CMRO_2_ surpassed CBF in the right putamen and supplementary motor area (Vafaee and Gjedde, 2004). Although our results regarding the relationship between rCMRO_2_ and rCBF are not identical to those between CMRO_2_ and CBF, the dissociated relationship between rCMRO_2_ and rCBF in the left PFC in response to ExL, with rCMRO_2_ being greater than rCBF, is in line with that observed in the previous PET study observed in the right putamen. The authors speculated that the relative changes in CMRO_2_ and CBF could be a guide for the postsynaptic processing of projections that are active while preparing for a neuronal task. After ExM termination, dissociated couplings between rCMRO_2_ and rCBF were identified in both sides of the PFC, with rCBF being greater than rCMRO_2_. This relationship is compatible as the magnitude of CMRO_2_ is smaller than increases in CBF in the sensory-motor cortex during simple somatosensory stimulation (Fox and Raichle, 1986) and in the striate cortex during visual stimulation (Mintun et al., 2002).

To observe CMRO_2_ and CBF simultaneously, single-session NIRS and arterial spin labeling with MRI have been previously demonstrated for CMRO_2_ measurement in rats and mice (Hashem et al., 2020). NIRS alone has also been used to measure CMRO_2_ in newborn piglets by combining two near-infrared techniques: diffuse correlation spectroscopy and TRS NIRS (Tichauer et al., 2006; Verdecchia et al., 2013). However, in these studies, CBF was determined using an invasive manner that involved the injection of indocyanine green as an intravascular tracer. These alternative methods to measure CMRO_2_ and CBF pose a great concern; however, to explore rCBF and rCMRO_2_ directly, future studies applying simultaneous TRS and PET measurements are required.

### 4.4 Limitations

The small sample size, possible selection bias, and several methodological limitations of our study do not allow us to draw generalizable conclusions. Other limitations are described as follows.

• For a constant power law relationship between changes in CBF and CBV investigating rhesus monkeys and newborn piglets (Grubb et al., 1974; Brown et al., 2003), Ito et al. demonstrated the relationship between CBF and CBV in response to hyper- and hypocapnia in humans as CBV = 1.09 × CBF^0.29^ (Ito et al., 2003). However, because the relationship between the changes in CBF and CBV differs depending on the stimulation applied, this relationship during exercise also differs from that observed via the cerebrovascular response to CO_2_ induced by inhalation or hyperventilation. To the best of our knowledge, simultaneous measurements to examine changes in CBF and CBV in response to exercise have not been reported using PET. Dual measurements of CBF and CBV obtained by the simultaneous application of PET and TRS will clarify this relationship when changes in CBF are evoked by exercise.
• To minimize the effect of extra-cerebral components, we selected TRS. Although appropriate, during exercise when skin blood flow increases, NIRS signals would be contaminated by extra-cerebral effects. Accordingly, it should be noted that the tNIRS-1 applies the diffusion equation for the semi-infinite homogeneous medium instead of the two-layer data fitting analysis head model, previously used to discriminate and quantify absolute extra-cerebral and cerebral hemoglobin concentrations during incremental physical exercise (Auger et al., 2016).
• Another limitation is that arterial blood sampling was not performed to measure HGB and SaO_2_, which is necessary to detect CBV and calculate CMRO_2_ using the estimated CBF. Additional research using arterial blood sampling is required to validate and complement our results.
• When using NIRS systems, artifacts caused by head movement and sweat during prolonged exercise should be considered, especially during high-intensity exercise. In our data processing, we carefully observed and removed artifact-induced errors. To detect the precise location in the PFC, additional studies involving MRI can clarify the area of NIRS data collection.

### 4.5 Summary

In summary, we examined cerebral oxygenation in the PFC and estimated the relationship among rCMRO_2_, rCBV, rCBF, and rOEF during low- and moderate-intensity cycling exercises. Low-intensity cycling exercise did not evoke increases in ScO_2_ and [HbT] in the PFC but did induce a decrease in ScO_2_ in the left PFC at 3 min after exercise initiation, which was reflected as the dissociated relationships between rCMRO_2_ and rCBV and rCMRO_2_ and rCBV. When exercise intensity increased, there was an increase in cardiovascular variables, which was accompanied with an increase in perceived exertion. There was also an increase in ScO_2_ and [HbT], and a dissociated relationship between rCMRO_2_ and rCBV and rCMRO_2_ and rCBF in the PFC was identified. Alterations in the relationship between cerebral oxygen metabolism and perfusion in the PFC could be caused by exercise intensity and the duration of physiological stimulation, which could be reflected in the functional role of the PFC during exercise.

## Data Availability

The original contributions presented in the study are included in the article/Supplementary Materials; further inquiries can be directed to the corresponding author.

## Supplementary material

SUPPLEMENTARY FIGURE S1 Changes in cardiovascular, respiratory variables and cerebral oxygenation variables obtained using near-infrared spectroscopy for the prefrontal cortex during and after a constant load of 17-minutes of low-intensity followed by 3-minutes of moderate-intensity constant-load cycling exercise for all participants (n = 12). **(A)** Heart rate and ScO_2_, **(B)** Heart rate and HbT, **(C)** P_ET_CO_2_ and ScO_2_, **(D)** P_ET_CO_2_ and HbT, **(E)** Relative $\dot{V}$ O_2_ and ScO_2_ and **(F)** Relative $\dot{V}$ O_2_ and ScO_2_. Data were averaged over 15 s for each time point. Changes in ScO_2_ for the left (red) and right (brown), and HbT for the left (green) and right (blue) prefrontal cortex are depicted. The thick bars below the NIRS parameter changes indicate the periods of the exercise session of light (gray) and Moderate intensity (black). $\dot{V}$ O_2_, pulmonary oxygen consumption; P_ET_CO_2_, end-tidal carbon dioxide; ScO_2_, cerebral hemoglobin oxygen saturation; HbT, total hemoglobin concentration in the brain tissue.
