# Supplementary figures and images for: Dissociated coupling between cerebral oxygen metabolism and perfusion in the prefrontal cortex during exercise: a NIRS study
Source: Front Physiol. 2023 Jul 25;14:1165939. doi: 10.3389/fphys.2023.1165939 (PMC10411551; doi:10.3389/fphys.2023.1165939)

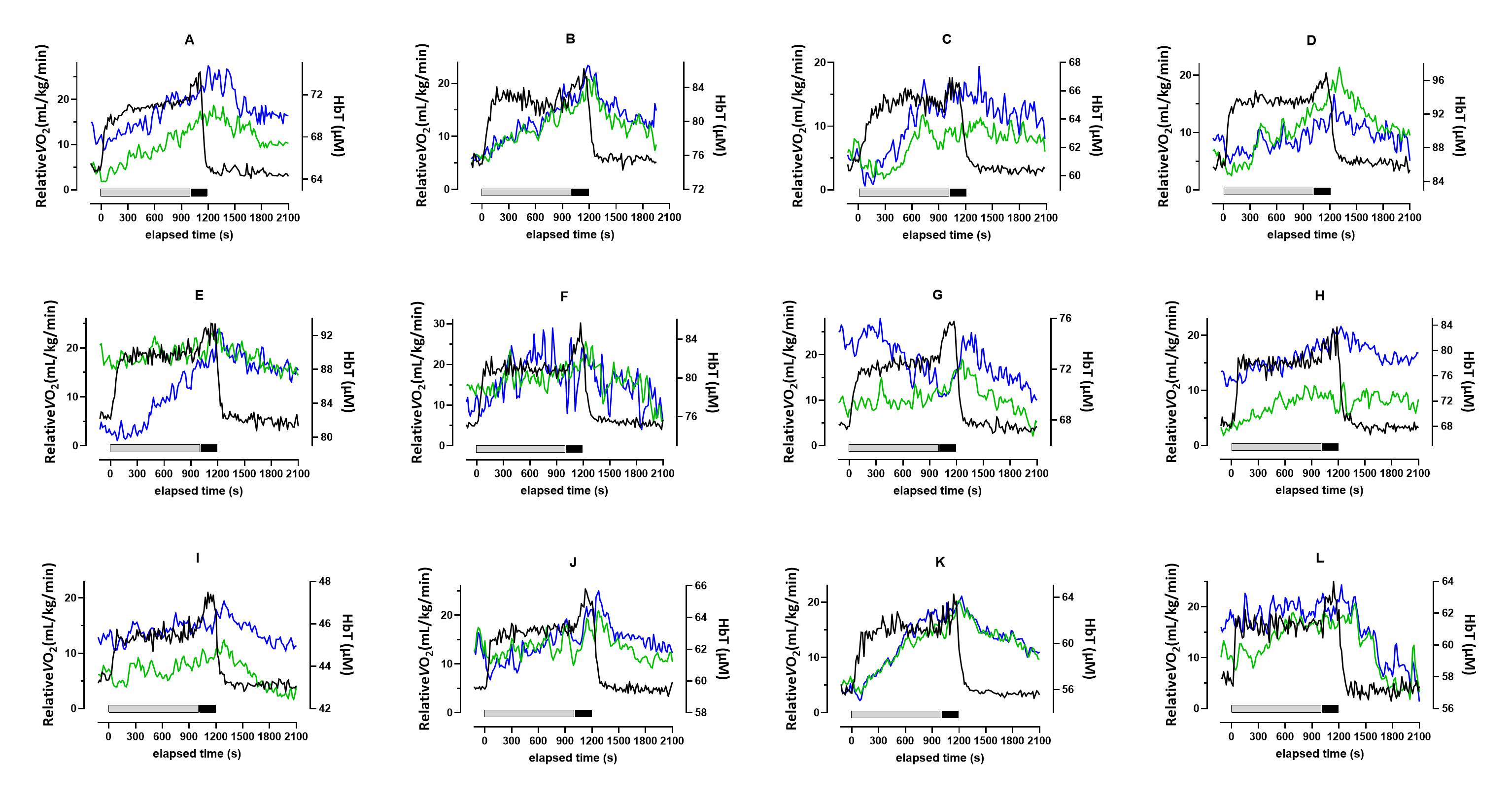

Supplement: Supplementary file 1 [file Image6.TIF]

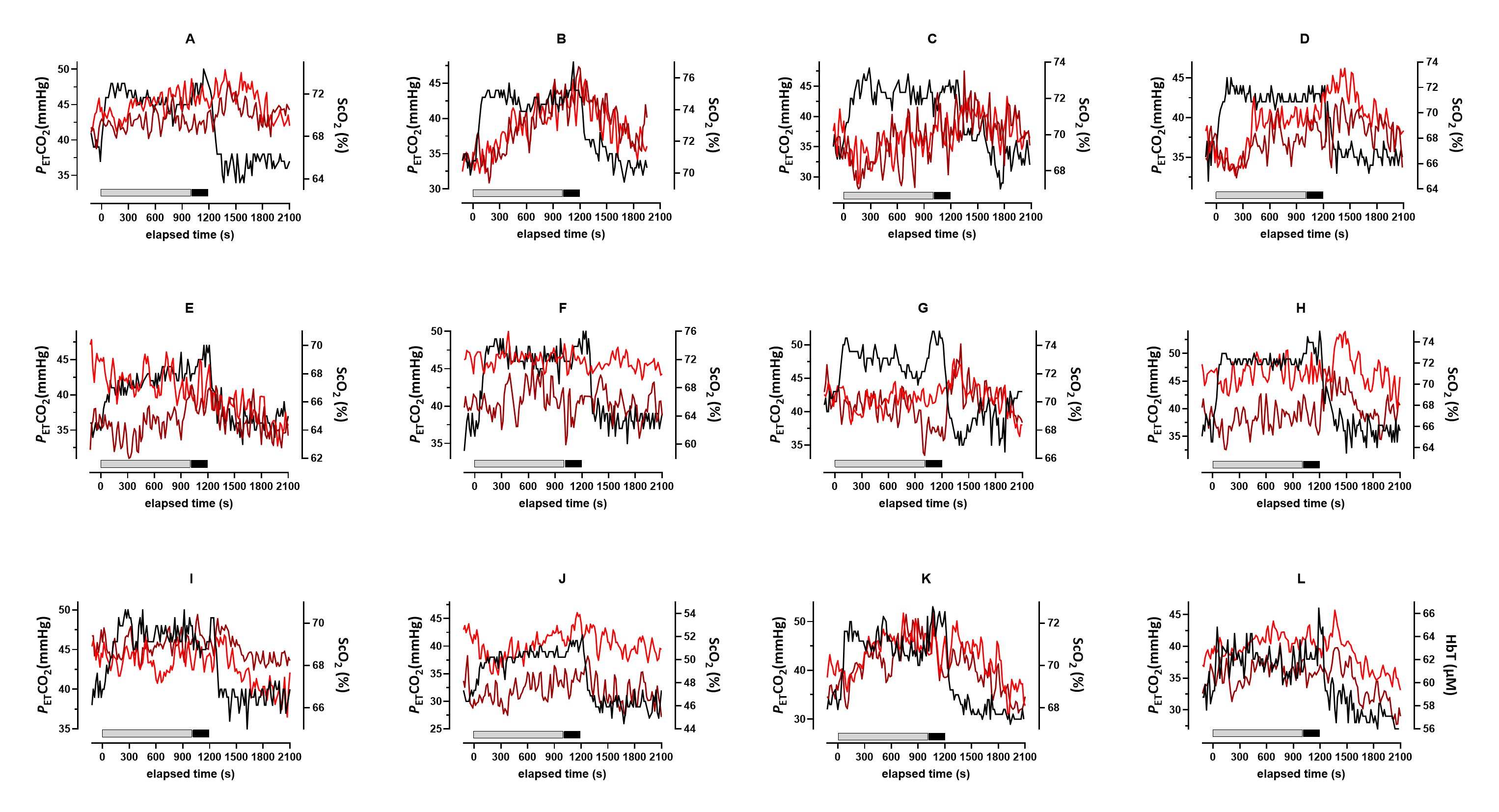

Supplement: Supplementary file 2 [file Image3.TIF]

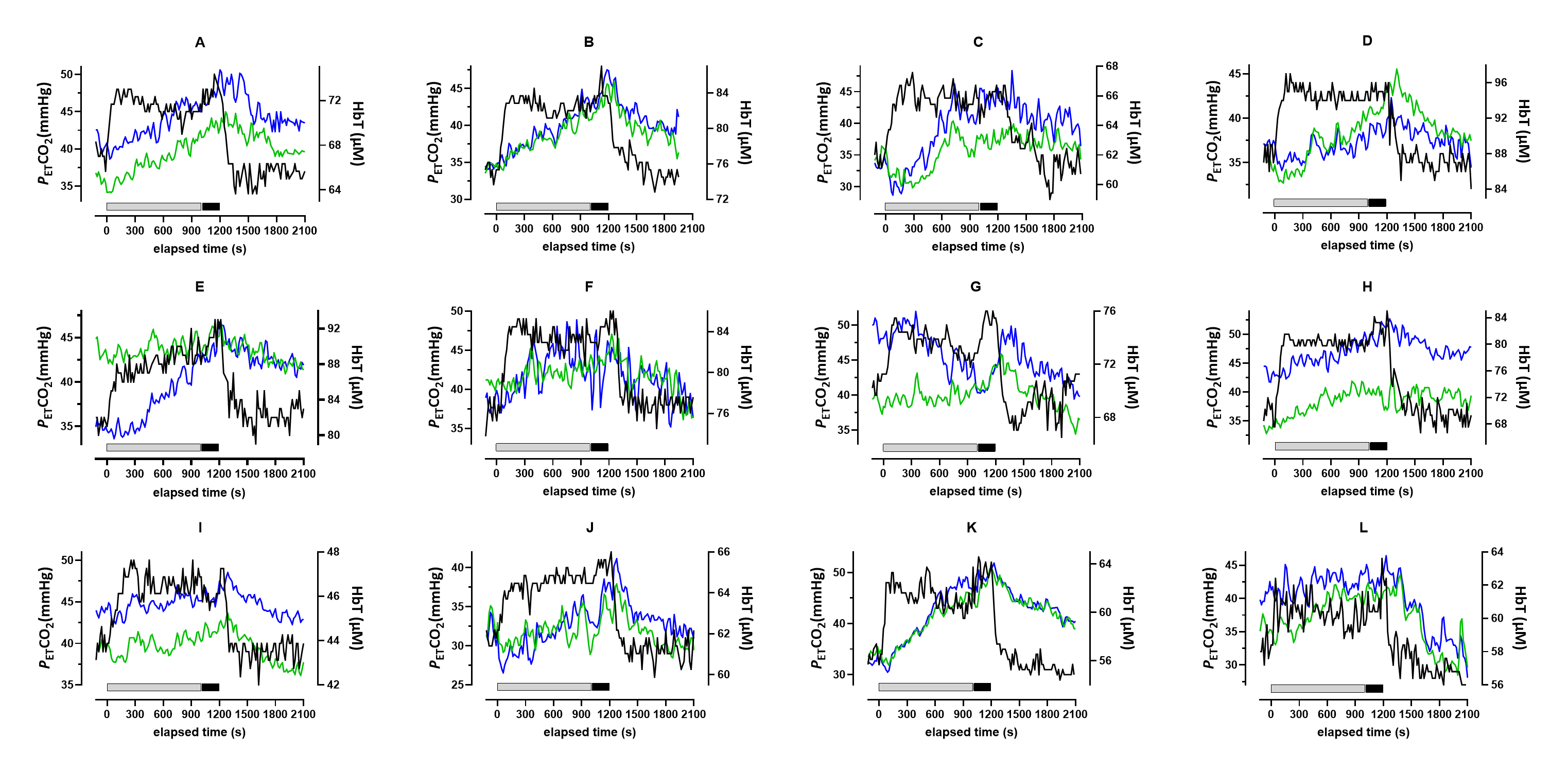

Supplement: Supplementary file 3 [file Image4.TIF]

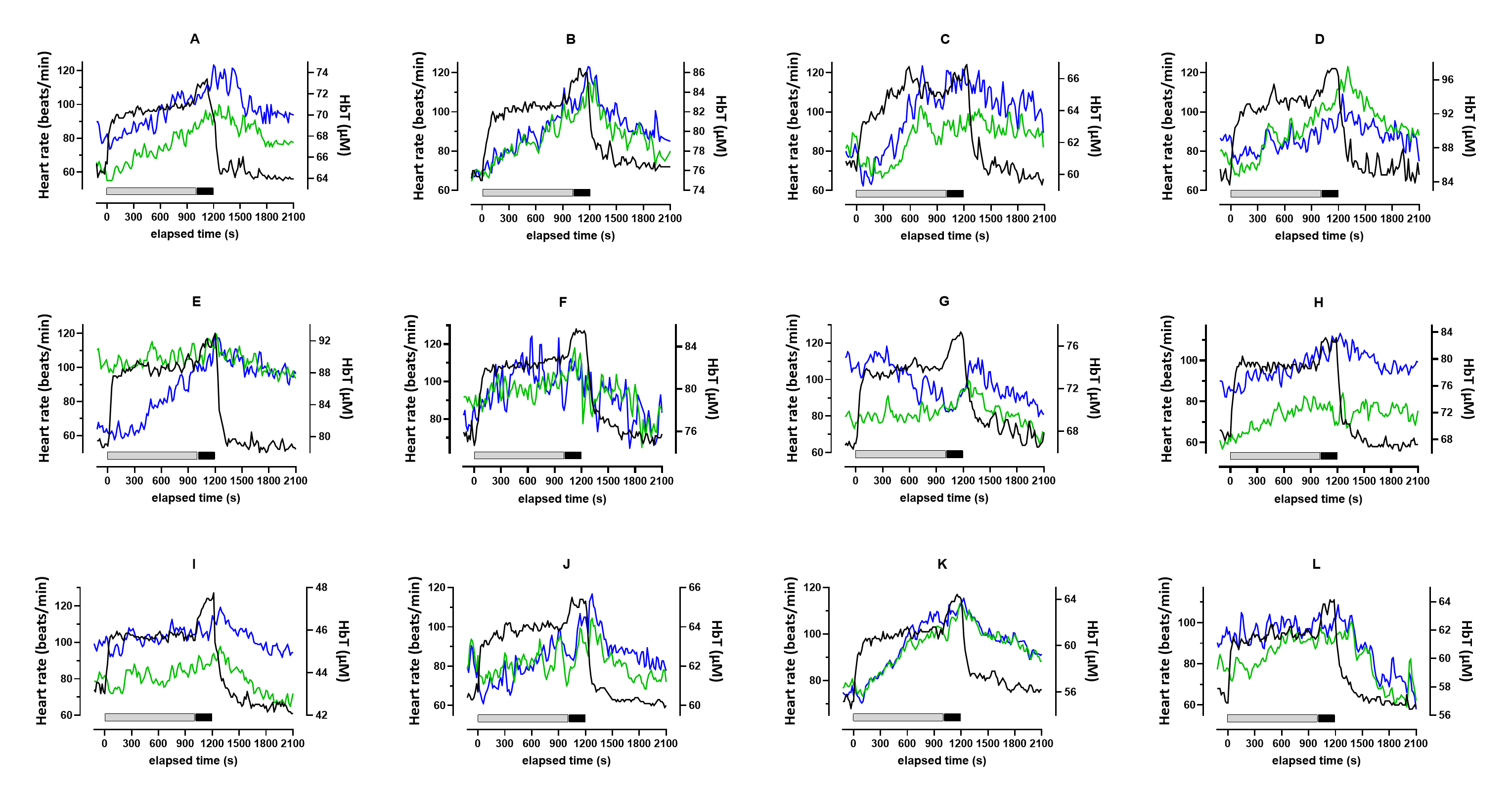

Supplement: Supplementary file 4 [file Image2.TIF]

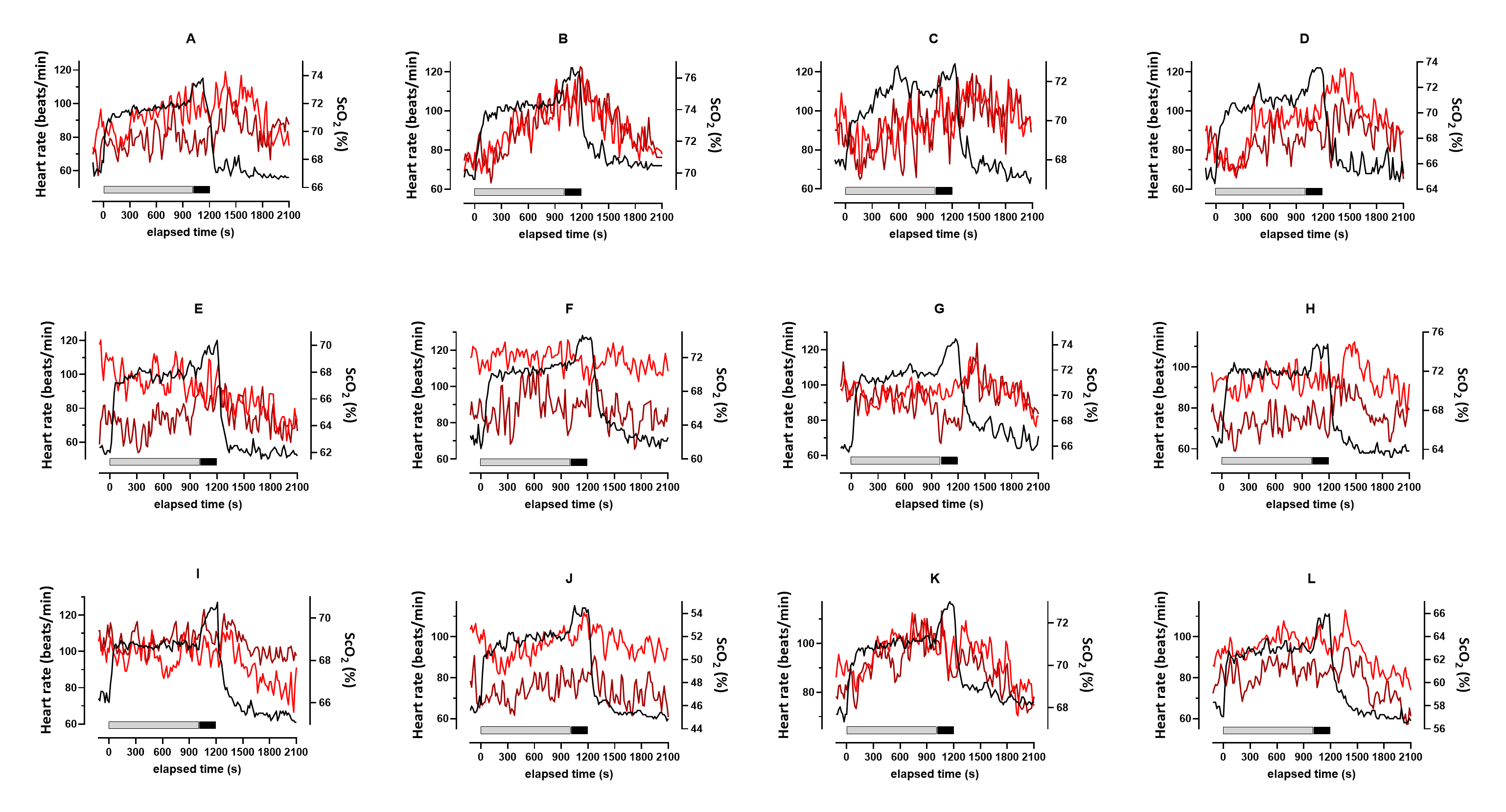

Supplement: Supplementary file 5 [file Image1.TIF]

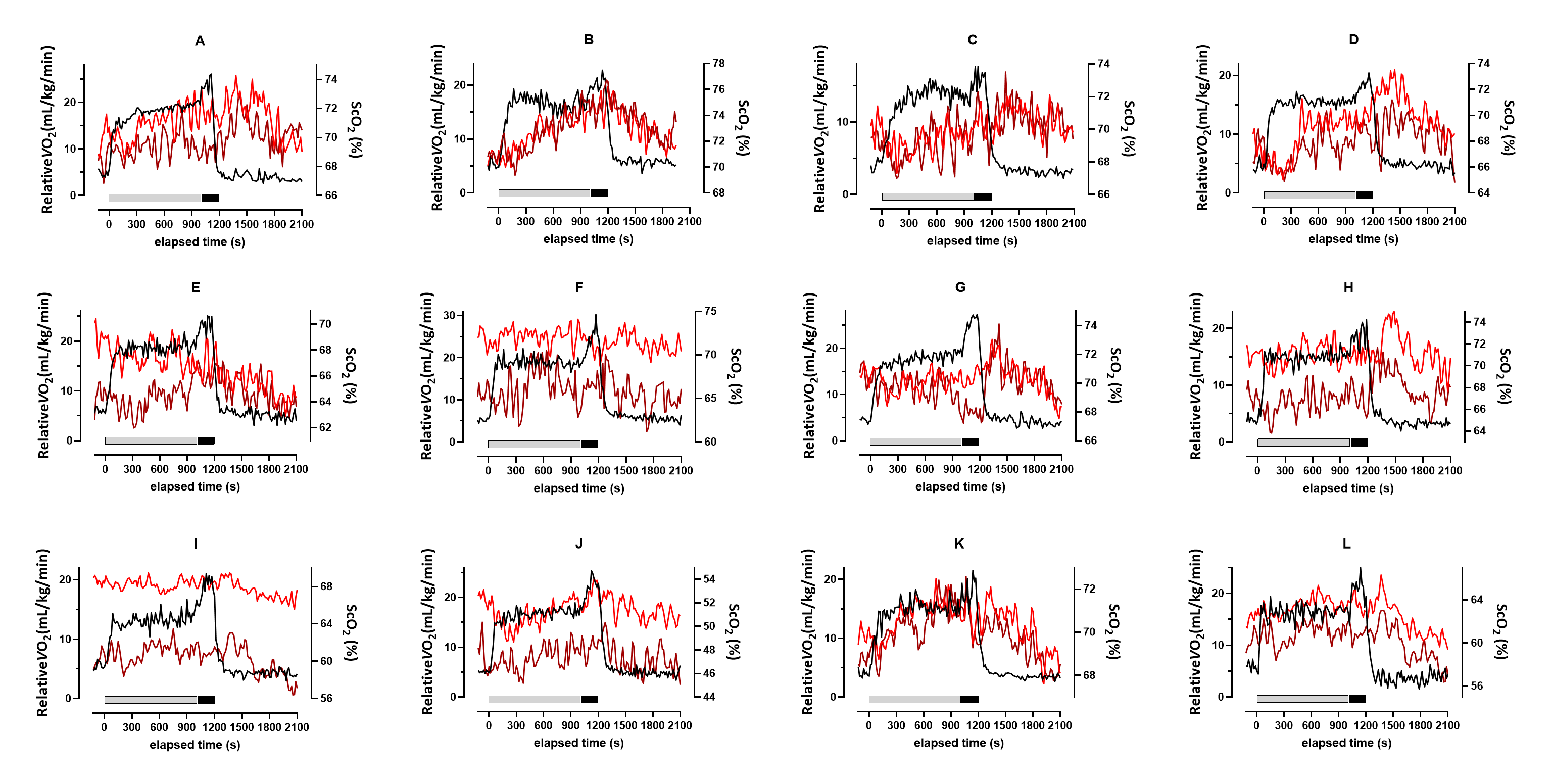

Supplement: Supplementary file 6 [file Image5.TIF]
